# Incidence and prevalence of neovascular age-related macular degeneration: 15-year epidemiological study in a population-based cohort in Finland

**DOI:** 10.1080/07853890.2023.2222545

**Published:** 2023-06-12

**Authors:** Ida Korva-Gurung, Anna-Maria Kubin, Pasi Ohtonen, Nina Hautala

**Affiliations:** a Department of Ophthalmology, PEDEGO Research Unit and Medical Research Center, University of Oulu, Oulu, Finland; b Department of Ophthalmology, Oulu University Hospital, Oulu, Finland; c Research Service Unit, Oulun yliopistollinen sairaala, Oulu, Finland; d The Research Unit of Surgery, Anesthesia and Intensive Care, Oulu University Hospital and University of Oulu, Oulu, Finland

**Keywords:** Age-related macular degeneration, ageing population, prevalence, incidence

## Abstract

**Background/objectives:**

Neovascular age-related macular degeneration (nAMD) is a common cause for visual impairment in the ageing population. An increasing number of nAMD patients causes significant health burden, although intravitreal anti-VEGF agents have revolutionized nAMD treatment during the past 15 years. We aimed to define incidence and prevalence of nAMD in different age-categories in the anti-VEGF era and to estimate the number of the individuals over 75 years of age in 2050.

**Patients and methods:**

We conducted an epidemiological study of the nAMD cohort (*n* = 2121) in a Finnish population of 410,000 inhabitants. Demographic and clinical data were gathered from Oulu University Hospital’s database during 2006–2020. The incidence and prevalence rates were calculated using population data from national registers. The three-year moving average of incidence of nAMD per 100,000 person years was estimated. Prevalence figures were calculated per 100,000 age-specific inhabitants.

**Results:**

The average age at the diagnosis of nAMD was 78土8 years, and 62% of the patients were women. The incidence of nAMD was 71 (95% CI 55–90) and 102 (95% CI 88–118) per 100,000 person years in 2006 and 2020, respectively. During 2006–2020, 1.2- and 2.4-fold increases in nAMD incidence were noted in 75–84 and in 85–96 age groups, respectively. In the oldest 75–84 and 85–96 age categories the nAMD prevalence was 2865/100,000 (3%, 95% CI 2665–3079) and 2620/100,000 (3%, 95% CI 2323–2956), respectively. The proportion of the inhabitants >75 years old is estimated to increase from 10% in 2020 to 17% by 2050.

**Conclusions:**

Our results indicate constant 1.2- and 2.4-fold increases in nAMD incidence during the past 15 years in age groups of 75–84 and 85–96 years, respectively, and 3% prevalence of nAMD in 2020. An almost two-fold increase in the ageing population by the year 2050 may also predict the trends in nAMD.

**KEY MESSAGES**
- The results of the current population-based study indicate 1.2- and 2.4-fold increases in the incidence of neovascular AMD (nAMD) during the last 15 years in the Finnish population aged 75–84 and 85–96 years and 3% prevalence of nAMD in 2020.
- An almost two-fold increase in the number of individuals over 75 years of age by the year 2050 is estimated, which may also predict the trends in nAMD.
- Intravitreal anti-vascular endothelial growth factor (anti-VEGF)- agents have revolutionized the treatment and prognosis of nAMD. Timely recognition and referral of nAMD patients to ophthalmologist can ensure vision-related functionality especially among the ageing population.

## Introduction

The global number of patients with age-related macular degeneration (AMD) has been estimated to rise from 196 million in 2020 to 288 million by 2040 [1]. The increase of prevalence and incidence of AMD in European countries by 15% and 75%, respectively, has been predicted in the ageing population until 2050 [2]. In Finland, inconsistent prevalence rates have been reported depending on the definition and classification of AMD [3,4]. Wong et al. have reported a current 12% prevalence of any AMD including both dry AMD and nAMD worldwide, whereas 28% prevalence of any AMD was reported in Europe [2]. AMD mainly affects the individuals over 75 years of age, and thus, the age-specific estimates for AMD incidence and prevalence are needed, particularly in cases of neovascular AMD (nAMD) requiring treatment.

AMD, and especially nAMD, is the main cause of visual impairment and blindness in developed countries [2,5,6]. According to the Finnish Register of Visual Impairment and the Finnish Institute for Health and Welfare, AMD causes up to 41% of the visual impairment in the entire population and 58% of the visual impairment in individuals over 65 years of age (https://www.nkl.fi). Accordingly, AMD was the cause of partial visual loss or blindness in 57–59% of the cases of visual impairment in the United Kingdom in 2007–2008 [7]. Advanced age, genetics and tobacco smoking predispose to AMD, although the aetiology of AMD is still not fully understood [8–10]. Avoiding the known risk factors for AMD may, in part, prevent or slow the progression of dry AMD to nAMD.

During the last two decades, anti-vascular endothelial growth factor (anti-VEGF) drugs have revolutionized the treatment of nAMD, and today these agents form a standard treatment protocol for nAMD [11,12]. Management of nAMD includes close monitoring and recurrent intravitreal anti-VEGF injections and requires substantial healthcare resources. The widespread clinical introduction of anti-VEGF therapy, however, has been suggested to be a reason for almost 30% decline in the age-standardized prevalence of blindness due to AMD during the years of 1990–2020 [13]. In the present study, we aim to determine the changes in age- and gender-specific incidence and prevalence of nAMD during the past 15 years. In addition, we estimate the increase in the number of people aged over 75 years by the year 2050, to consider the burden of nAMD treatment in the future healthcare planning.

## Patients and methods

This population-based epidemiological study was performed on all patients (*n* = 2121) with nAMD during 2006–2020 treated at the Oulu University Hospital, which is responsible for tertiary care for a population of approximately 410,000 inhabitants. In Finland, municipalities are responsible for organizing and financing health care, and every citizen is entitled to adequate health services. Specialized healthcare refers to examinations and treatments arranged in hospitals in specialized fields, such as ophthalmological examinations and treatment of eye diseases, including nAMD. Access to ophthalmic treatment is managed according to certain criteria of clinical pathways, and it requires a referral.

The hospital’s electronic patient database was used to search for the nAMD patients treated with intravitreal anti-VEGF agents by using the ICD-10 (International Classification of Diseases) diagnosis code for nAMD (H35.31). All patients with a nAMD diagnosis had undergone a comprehensive ophthalmic examination, evaluation of best-corrected visual acuity and fundus imaging ((fundus photography, fluorescein angiogram, optical coherence tomography (OCT), optical coherence tomography angiography (OCT-A)) based on discretion of the treating physician and the availability of the imaging techniques during the follow-up period. The cases of dry AMD, polypoidal choroidal vasculopathy (PCV), myopic degeneration or other retinal disorders, such as DME, treated with anti-VEGF injections were excluded from the study. The treatment and follow-up of nAMD patients in Finland is performed according to the Current Care Guidelines for AMD [12]. The patients with nAMD have been treated with intravitreal anti-VEGF injections since 2006, but the treatment protocols have improved from single injections at four to six week-intervals to three to six consecutive injections at fixed intervals depending on the treatment protocol. In Finland, bevacizumab is commonly the first-line choice for the treatment of nAMD. Demographic and clinical data was collected and included parameters for time of the diagnosis, gender and age at the onset of nAMD. The characteristics of the participants, annual incidence and prevalence of nAMD in age groups of 44–64, 65–74, 75–84 and 85–96 years and between the genders were the main outcome measures. The age of the patients varied from 44 to 96 years. The 10-year age brackets were used in the patients aged over 65 years, but in the youngest age group the age bracket was larger due to the small (only 8% of all) number of patients. The age categories were used to describe the differences in the rates of nAMD in different age-groups. The approximate number of elderly populations in 2050 was calculated to appraise the future burden for nAMD.

The population data for years 2006–2020 and 2050 was offered by Statistics Finland. The three-year moving average was used for calculations of incidence of nAMD per 100,000 person years among adults in each age group. The prevalence figures were calculated per 100,000 age-specific inhabitants. Ninety-five % confidence intervals (95% CI) are presented for incidence and prevalence. The patients that moved to another hospital district or became deceased, were excluded at the end of the year. The study followed the tenets of the Declaration of Helsinki and it was conducted with the approval of the Oulu University Hospital Research Committee (permission ID 221/2016). A written informed consent was obtained from the participants at the time of the clinical ophthalmic evaluation.

SPSS for Windows (IBM Corp. Released 2019. IBM SPSS Statistics for Windows, Version 26.0. Armonk, NY: IBM Corp) was used for data analysis.

## Results

A total of 2121 patients with nAMD were included in the population-based study cohort. Out of the 2121 patients, 1320 (62%) were women and 801 (38%) were men. According to the data from Statistics Finland over 98% of the population aged over 75 years in Finland are Finnish background (white Caucasian by ethnicity). The average age at the nAMD diagnosis was 78 ± 8 years. During the years 2006–2020, the average age at the nAMD onset ranged from 76 to 79 years. 49% of the nAMD patients were in the age-group of 75–84 years and 20% in the oldest group of 85–96 years. Women were a majority except for the age groups under 74 years of age. The gender distributions in various age-groups are presented in Table 1.

**Table 1.** Demographics and gender distribution of the study patients.

	Female (*n* = 1320, 62%)	Male (*n* = 801, 38%)	Total (*n* = 2121)	*p* Value
Age group, years				<.001^a^
44–64, *n* (%)	80 (6)	80 (10)	160 (8)	
65–74, *n* (%)	282 (21)	217 (27)	499 (24)	
75–84, *n* (%)	670 (51)	363 (45)	1033 (49)	
85–96, *n* (%)	288 (22)	141 (18)	429 (20)	
Mean age (SD)	78 (8)	77 (9)	78 (8)	<.001^b^

^a^Pearson Chi-square test.

^b^Student’s *t*-test.

**Figure 1.**
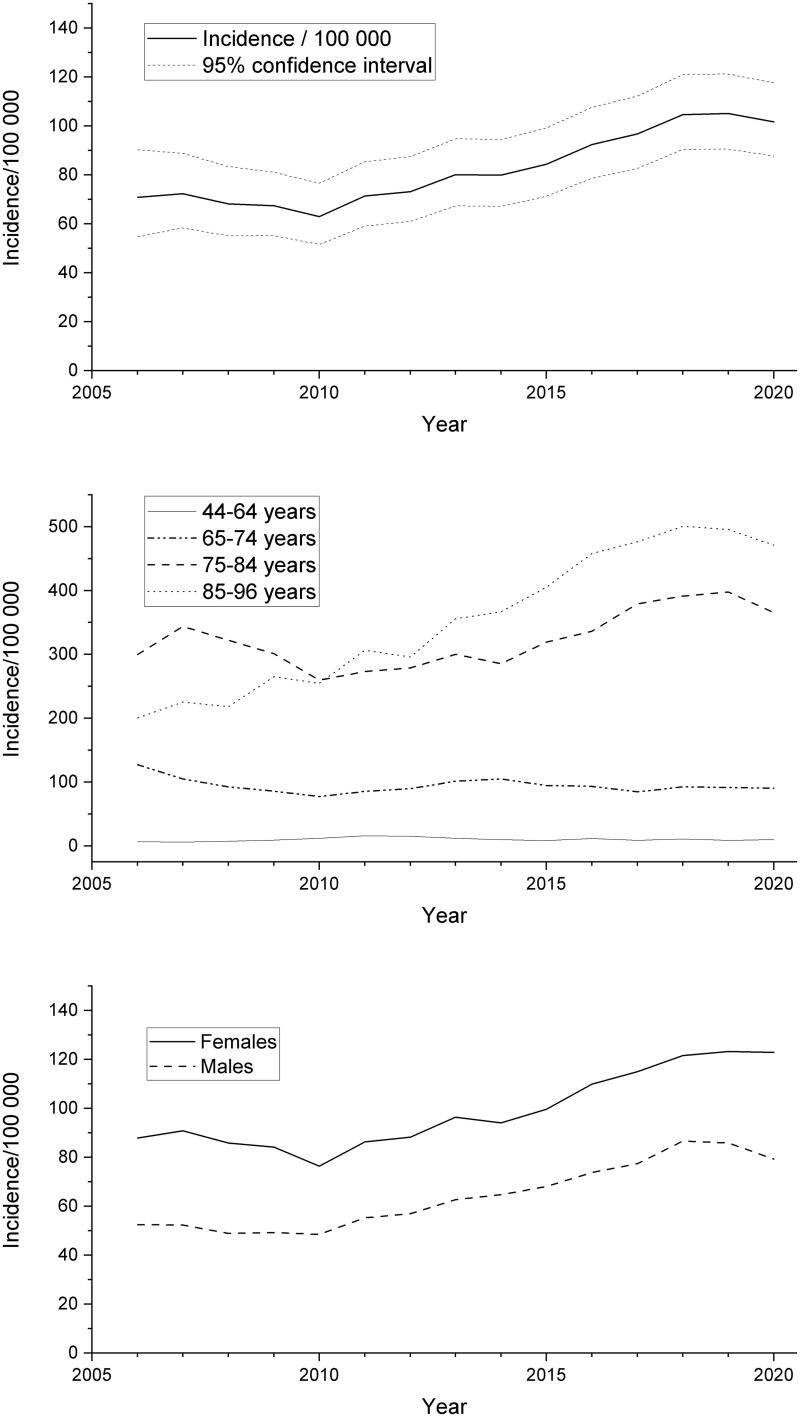
Overall incidence of neovascular age-related macular degeneration (nAMD) calculated as three-year moving average per 100,000 person years (above), age-adjusted rates for nAMD incidence (middle) and gender distribution (below) in 2006–2020.

The incidence of nAMD in the age group of 44–96 years was 71 (/100,000 person years) in 2006 (95% CI 55–90) and 102 in 2020 (95% CI 88–118) (Figure 1). In the age group of 75–84 years, the incidence increased 1.2-fold from 300 in 2006 to 366 in 2020. The increase in the age group of 85–96 years was 2.4-fold from 200 in 2006 to 471 in 2020. The incidence of nAMD in women was almost two-fold greater compared to males at the same age. In the oldest age groups and in the male gender, a slight plateau in the incidence of nAMD was documented during the last years of the study (Figure 1).

**Figure 2.**
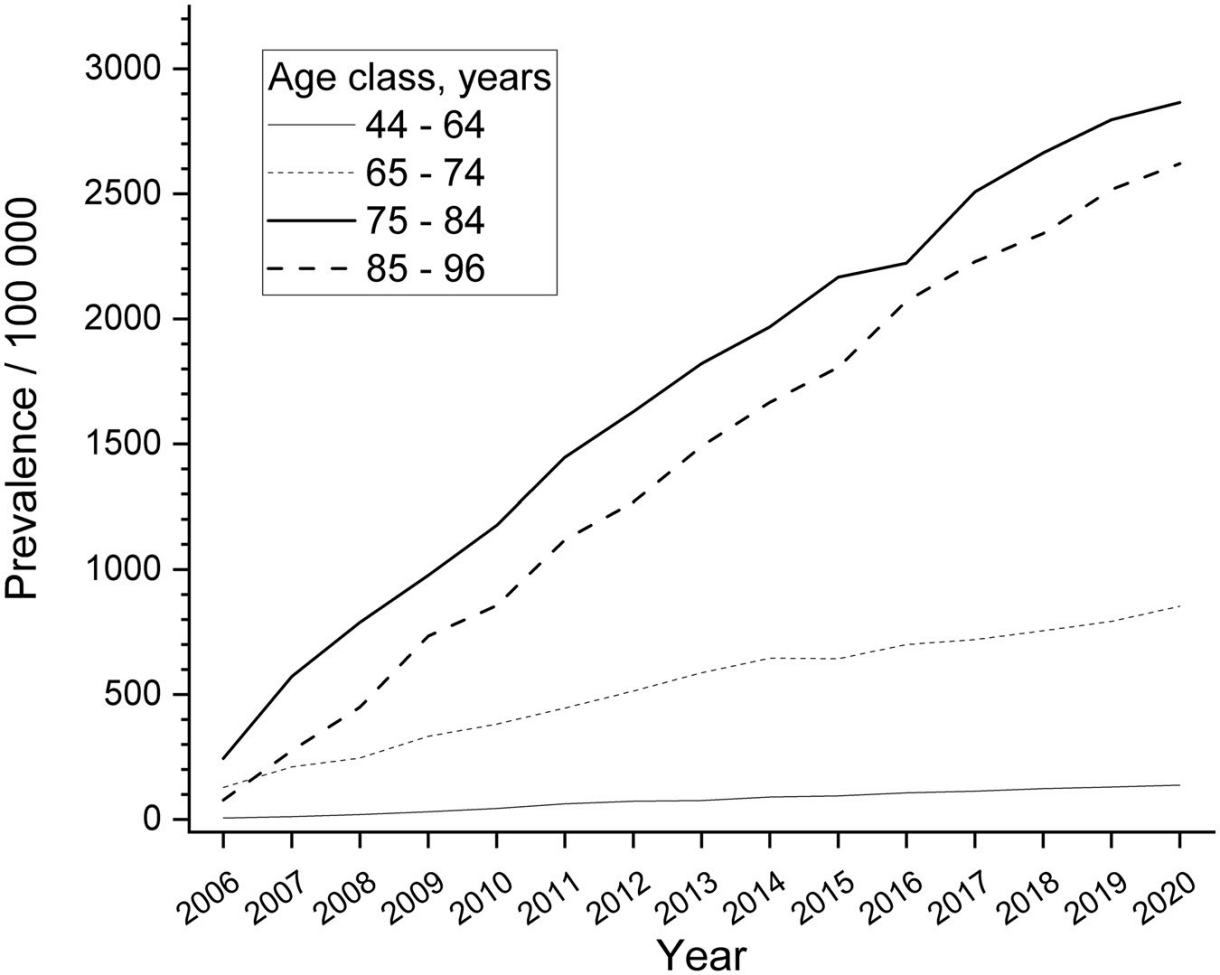
Age-group specific prevalence of neovascular age-related macular degeneration (nAMD) in 2006–2020. The prevalence figures were calculated per 100,000 age-specific inhabitants.

The prevalence of nAMD was 862 (/100,000 inhabitants) (95% CI 821–906) in adults aged 44–96 years in 2020. In the older age groups of 75–84 and 85–96 years the 3% prevalence was documented (2865/100,000 inhabitants, 95% CI 2665–3079 and 2621/100,000 inhabitants, 95% CI 2323–2956, respectively). The prevalence of nAMD increased enormously in all age groups during the years 2006–2020: from 6 to 138 in the age group of 44–64, from 128 to 853 in the age group of 65–74, from 244 to 2865 in the age group of 75–84 and from 78 to 2621 in those aged 85–96 years (Figure 2).

The number of all inhabitants exceeding 75 years of age in Finland is estimated to increase almost 1.7-fold from 2020 to 2050, *n* = 550,000 (10% from the whole population) and *n* = 918,000 (17%), respectively.

## Discussion

Our results demonstrate constant 1.2- and 2.4-fold increases in the incidence of nAMD in the elderly population aged 75–84 and 85–96 years during the last 15 years in Finland and 3% prevalence of nAMD in 2020. This increasing trend of AMD agrees with the estimated increase in the number of AMD patients worldwide from 196 million in 2020 to 288 million by 2040 [1,14,15]. The previous studies from European populations have reported 16–28% prevalence of any AMD [1,2,16]. Prior Finnish studies have revealed that any AMD affects 12% of the inhabitants ≥ 65 years and 27% of those ≥ 85 years [2,4]. These numbers including the prevalence rates of both dry AMD and nAMD cannot be compared to the results of the present study presenting the prevalence of nAMD only. The discrepancies in the prevalence rates may also reflect the differences in the underlying classification system of AMD [17]. In contrast to our results, several previous studies present only the best estimates of the prevalent and incident cases of nAMD in the absence of representative population-based data.

The study participants aged 75 years or older were mostly affected by nAMD and the prevalence rate of nAMD seems to vary greatly depending on the age of the participants. Accordingly, Wong et al. reported 16%, 25% and 33% prevalence rates for any AMD in patients aged 60–69, 70–79 and 80–84 years, respectively [1]. In addition, the estimated prevalence of late AMD was only 0.03% in those aged 45–49 years but increased up to 5% in the oldest age group of 80–84 years [1]. Comparably, a recent meta-analysis revealed 0% prevalence of any late AMD in populations aged 50–55 years and 20% prevalence rate of any late AMD in the individuals aged ≥90 years [18]. Also in an earlier Finnish study, the highest prevalence of AMD was noted in the individuals aged 85 years or older [3]. In our study, the incidence of nAMD was also higher in female participants compared to age-matched males, in accordance with the previous study [18]. The variation in the prevalence rates of nAMD depends greatly on the age and gender and further underlines the importance of age-specific pooled prevalence estimates. The cross-over of incidence figures between the age groups of 75–84 and 85–96 might be explained by possible under-reporting of nAMD in the oldest age group during the early years of anti-VEGF treatment and latter increased clinical evidence of safety and efficacy of anti-VEGF injections also in the oldest age group. Interestingly, a slight decline in the incidence rates for nAMD were noted recently in our study, particularly in the oldest age-groups. This may represent the true plateau in the nAMD incidence in the ageing population or reflect the effect of the COVID-19 pandemic-related lockdown period in 2020. Recent studies have documented reduced ophthalmic presentations and diagnoses due to delays in patients seeking appropriate medical attention during COVID-19 related outbreak [19–21].

There are some limitations and possible sources of bias in our study. First, the diagnosis of nAMD in the study participants altered through the study years from 2006 to 2020 due to developments in non-invasive diagnostic imaging, particularly OCT and OCTA. Secondly, in the first years of the study, relatively low nAMD incidence and prevalence rates may have been affected by non-established status of anti-VEGF treatment for nAMD at that time, causing underestimation of incidence and prevalence figures. In addition, the study included participants from a single hospital area. However, the geographical area was large, contained several communities and the treatment of nAMD patients in the area was centralized in the Oulu University Hospital. In our opinion, 2121 study patients represented the typical patient with nAMD in Europe. We consider the real-life population-based setting as a strength of the current study, among the extension of the study time to the past 15 years covering the era of anti-VEGF treatment for nAMD. Moreover, we particularly aimed to evaluate the rates for nAMD, which today is among the ophthalmic disorders causing the most burden for health care providers in all industrialized countries with ageing population. However, not all patients diagnosed with nAMD are under constant active treatment after stabilization of the disease. To date, there is still no commonly used treatment for dry AMD in daily clinical practice, although the complement C3 inhibitor pegcetacoplan has been recently reported to reduce the growth of geographic atrophy in patients with dry AMD [22–24].

Our results suggest that a 1.7-fold increase from 550,000 to 918,000 inhabitants in the age groups mostly affected by nAMD (≥75 years) is expected until the year 2050 which may compare to the increasing rate of nAMD patients requiring continuous treatment for nAMD. At present, almost 70 million people in Europe are affected by AMD, and along with the ageing of the population the number of patients with AMD is expected to increase by 15%, and a 1.7-fold increase in the incidence of late AMD can be expected by 2050 [2]. Thus, additional healthcare resources and novel treatment strategies including new drugs with more sustained effect might be needed due to the increasing number of nAMD patients requiring constant monitoring and treatment in the future.

Currently, AMD is a leading cause of vision impairment and blindness in the elderly in industrialized countries [2,5]. With the advent of anti-VEGF-therapy during the past two decades, the visual outcome of patients with nAMD has been improved and measurable reductions of legal blindness incidence have emerged [11]. Today, approximately 20 million annual injections of anti-VEGF agents are given globally, and the costs of 350 billion US$ have been assessed for nAMD treatment worldwide (www.brightfocus.org/sources-macular-degeneration-facts-figures). According to our results, an almost two-fold increase in the number of the ageing population and potentially increased number of individuals with nAMD during the next decades is expected to further increase the burden for eye health care providers. Further research is needed to address sustained alternatives for the treatment and potential ways to prevent nAMD to relieve the future workload. The current age-specific population-based real-life data may be exploited for factual and proactive healthcare resource planning. To ensure vision-related functionality in ageing by effective treatment, recognition of nAMD and timely referral to an ophthalmologist by physicians treating older individuals is of importance.

## Data Availability

The data that support the findings of this study are available from the corresponding author, NH, upon reasonable request.
